# Different time-courses for consolidating information in working memory and long-term memory

**DOI:** 10.3758/s13423-026-02883-x

**Published:** 2026-03-09

**Authors:** Ana Rodríguez, Alessandra S. Souza, Klaus Oberauer

**Affiliations:** 1 Department of Psychology, University of Zurich, Zurich, Switzerland; 2 Faculty of Psychology and Education Sciences, University of Porto, Porto, Portugal

**Keywords:** Short-term consolidation, Working memory, Long-term episodic memory, Continuous reproduction

## Abstract

The formation of memory representations requires the transformation of perceptual information into stable traces via short-term consolidation. In this study, we measured the time-course of forming color–object associations in working memory (WM) and in long-term memory (LTM), and examined whether a single consolidation process supports both memory systems. Across three experiments, we estimated consolidation time-accuracy curves in WM and LTM by varying the free time to consolidate the information from 300 to 8,050 ms in a continuous reproduction task. We found that both WM and LTM showed a consolidation onset at approximately the same time, but the process followed distinct trajectories: WM consolidation progresses faster and reaches an asymptotic performance earlier compared to LTM consolidation. Our findings indicate that the creation of robust WM and LTM representations can run partially in parallel, but follows different time-courses in each memory system. This suggests that different processes may contribute to memory formation for immediate and later retrieval, and that a substantial contribution of LTM to performance in WM tests is more likely with slower presentation rates.

How rapidly are memories created? In this study, we examined the time-course of creating a stable memory representation for immediate and for later retrieval. Immediate access to goal-relevant representations relies on working memory (WM), a capacity-limited system. In contrast, long-lasting representations are supported by long-term memory (LTM), a system with potentially unlimited storage capacity. Though functionally distinct, WM and LTM are closely interrelated (Atkinson & Shiffrin, 1968; Cowan, 2008; Oberauer, 2009; Unsworth & Engle, 2007): The creation of stable WM representations contributes to the formation of LTM traces (Cotton & Ricker, 2021; Souza & Oberauer, 2017), and pre-learnt LTM traces can reduce WM load (Bartsch & Shepherdson, 2022; Sobrinho & Souza, 2023).

The creation of a stable WM representation is assumed to occur via *short-term consolidation* (Jolicœur & Dell’Acqua, 1998; Nieuwenstein & Wyble, 2014; Ricker et al., 2018; Ricker & Hardman, 2017)—a process that requires central attention for some time to be completed (Jolicœur & Dell’Acqua, 1998; Stevanovski & Jolicœur, 2007). It starts after the perceptual information has been encoded (Stevanovski & Jolicœur, 2007) and continues after it has been masked (Nieuwenstein & Wyble, 2014; Ricker & Cowan, 2014). Short-term consolidation has been studied by varying the free time between memory items. More time is assumed to reduce attentional competition between consolidation of each item (Ricker & Cowan, 2014; Souza & Oberauer, 2017), a benefit that asymptotes at around 500–1,000 ms (Nieuwenstein & Wyble, 2014; Ricker et al., 2018).

There are no estimates of the time-course for building a stable episodic LTM representation. Moreover, it is still an open question whether short-term consolidation is part of the formation of LTM traces or whether both consolidation processes run independently (for a review, see Cotton & Ricker, 2022). While previous research showed that longer consolidation time in WM improved subsequent recall for episodic LTM (Cotton & Ricker, 2021; Souza & Oberauer, 2017)—for a meta-analysis see (Hartshorne & Makovski, 2019)—benefits of free time may arise from different processes for each system.

The time-course for forming LTM representations is relevant for a further question: Researchers have argued that episodic LTM contributes to WM performance when WM capacity is exceeded (Bartsch & Oberauer, 2023; McCabe, 2008; Oberauer et al., 2017; Oberauer & Awh, 2022; Unsworth & Engle, 2006a, 2006b, 2007). However, it remains unclear whether episodic LTM representations could be created fast enough during the short encoding time in WM tasks to contribute substantially to WM performance.

As a first step to clarify the role of consolidation over the short and long term, we measured the time-course of establishing memory representations in WM and LTM. For this purpose, we varied the free time between items over a broad range of intervals and tested both systems using the same procedure. This allowed us to assess whether a shared consolidation process underlies the creation of a short and long-term representation. If so, we should observe a similar rate of improvement over time for both memory systems, which would be consistent with the proposal that rapidly formed episodic LTM traces contribute to WM when its capacity is exceeded. Conversely, if the rate is slower^1^ for episodic LTM, it would question the hypothesis that both systems rely on a single consolidation process, and it would place constraints on the circumstances in which new episodic traces contribute to performance in WM tests.

Four potential scenarios may clarify the interplay of WM and LTM consolidation: a) a shared initial consolidation process, followed by a slower one that further strengthens LTM traces; b) sequential consolidation, such that LTM consolidation starts after WM consolidation is completed (Cotton & Ricker, 2022); c) parallel consolidation (Cotton & Ricker, 2022); and d) cascading consolidation, in which LTM consolidation builds continuously on WM consolidation output (McClelland, 1979).

Across three experiments, we tested the role of consolidation time in a continuous-reproduction color–object task, where free time was varied within subjects across six levels (from 0 to 7,050 ms). We modeled the time-accuracy functions for each memory system to estimate when consolidation starts, the rate at which it proceeds, and when it asymptotes.

In Experiment 1, we differentiated WM and LTM based on both set size (3 vs. 20 items) and delay to the test (immediate vs. after a 1-min distractor task). In Experiment 2, we differentiated WM and LTM based only on delay while keeping set size constant at three items. Additionally, we randomly intermixed WM and LTM trials to prevent participants from anticipating the retrieval demands and thereby applying different maintenance strategies. Finally, in Experiment 3, we kept the design of Experiment 2 but changed the distractor task presented during the delay in LTM trials. In Experiments 1 and 2, the distractor task consisted of an unrelated orientation memory task that did not share features with the main task. We were concerned that this task did not fully eliminate representations of the object–color conjunctions in WM. Therefore, in Experiment 3, the distractor task consisted of a word-color binding task that created overlap and interference in the color domain, as in the verbal domain to maximize interference in WM and fully disrupt any remaining short-term trace, thereby increasing the likelihood that retrieval relied uniquely on LTM.

## General method

### Open practices statement

Materials, data, and analysis scripts for all experiments reported here are available at: https://osf.io/t8e3p/?view_only=1064bf607a724f47b77aeacb2d158fb3.

### Participants

Each experiment included an independent sample of 24 students (*M* = 22.6, *M* = 20.9, and *M* = 23.96 years old for Experiment 1, 2, and 3, respectively). Participants completed six web-based sessions, each lasting about 20–30 min, in exchange for course credit, a 50 Swiss-francs gift-card or 50 Swiss francs. Sample size was determined based on the necessary number to counterbalance the order of the experimental conditions across participants using a Latin square. The experimental protocol was in accordance with the ethics guidelines of the Institutional Review Board and did not require special approval.

### Materials and procedure

All experiments were programmed in Lab.js (Henninger et al., 2021). We used icons of concrete objects randomly chosen from a pool of 284 objects retrieved from Sutterer and Awh (2016). Each object was filled with one of 360 colors from a color wheel defined in the CIELab color space, centered on L = 70, *a* = 20, *b* = 38, with a radius of 55.

The objects were chosen at random at the beginning of each session and kept constant across trials, but their order and color varied randomly each trial. This was done to maximize proactive interference across WM trials, reducing the chance of LTM contributing to WM. For the LTM test, we used the same method to allow unconfounded comparison. In Experiment 1, each session included 13 WM and two LTM trials, using fixed sets of three objects for WM and of 20 objects for LTM trials. The task order was counterbalanced across participants. In Experiments 2 and 3, each session included the same set of 3 objects for both WM and LTM tasks, presented in intermixed trials (14 trials per task). Therefore, at encoding, participants could not anticipate whether the upcoming test would occur immediately or after a distractor-filled delay.

**Fig. 1:**
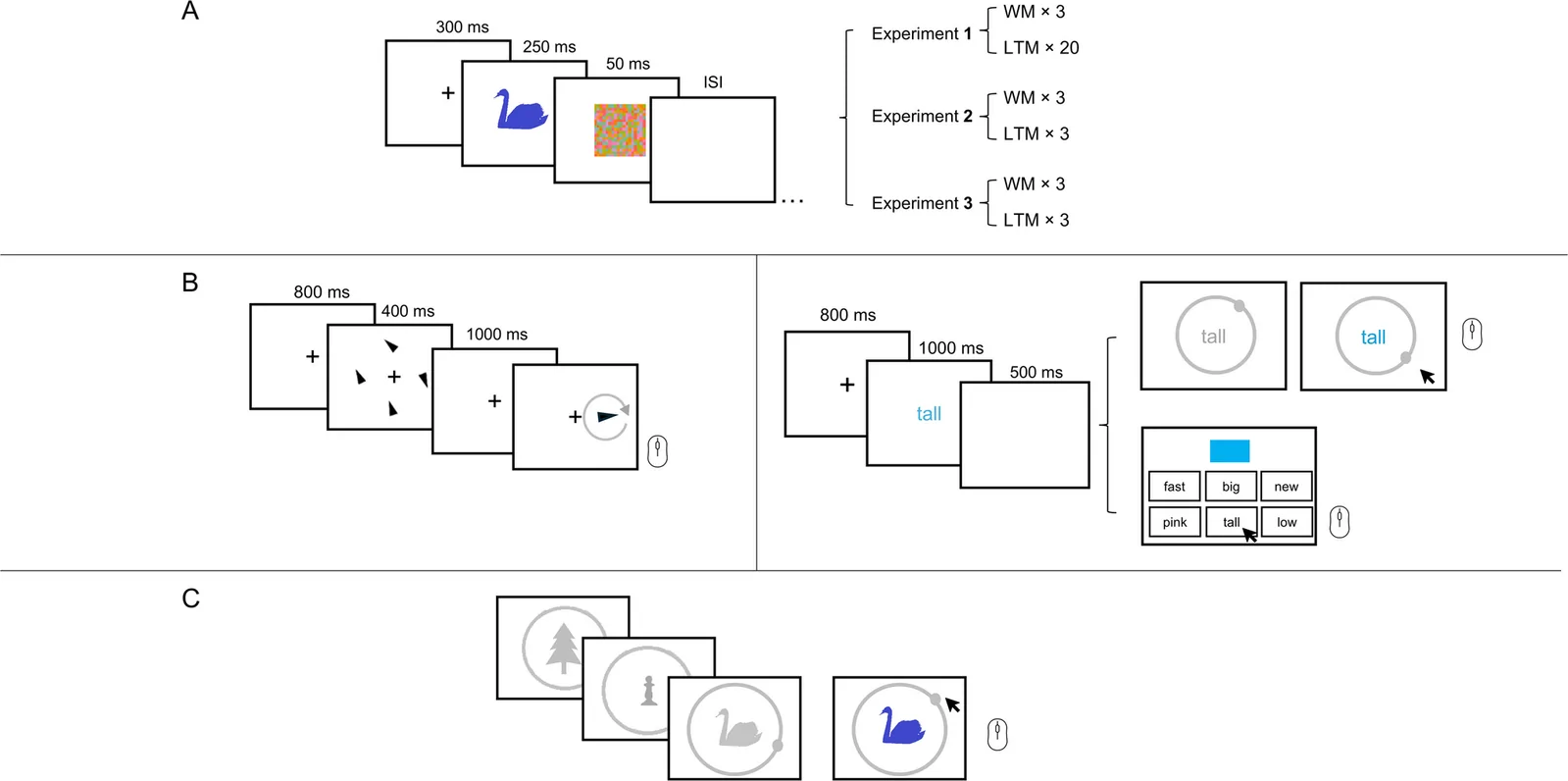
Flow of events of an experimental trial of the WM and LTM tasks. A Encoding phase. B Distractor task during the retention Interval for the LTM task: Experiments 1 and 2 (left panel), and Experiment 3 (right panel). C Test phase. ISI = interstimulus interval varied across six levels: 0, 250, 750, 1,750, 4,750, and 7,750 ms. Stimuli are not drawn to scale. (Color figure online)

As illustrated in Fig. 1A, each WM and LTM trial began with the presentation of a central fixation cross for 300 ms, after which a sequence of colored objects (size = 150 pixels) was displayed on three possible locations evenly spaced on an imaginary circle (radius = 100 pixels) centered in the middle of the screen (up, right, and left). The first stimulus position was always the upper position, and the subsequent ones followed in anti-clockwise fashion. Each object was presented for 250 ms, followed by a mask (size = 150 pixels; 50 ms) composed of randomly placed colored dots. After the mask, a blank interstimulus interval (ISI) of 0, 250, 750, 1,750, 4,750, or 7,750 ms followed, implementing the manipulation of consolidation time. The consolidation time is the time between the onset of one item and the onset of the next item (or of the distractor task or the memory test)—that is, the sum of the presentation time, the mask duration, and the ISI. In each session, a single ISI was implemented for all trials in both tasks. By not mixing stimuli with different ISI within a session, we avoided the problem of having representations with potentially different memory strength that competed with each other for retrieval.

In all experiments, for WM trials the sequential presentation of the objects was followed by an immediate color reproduction test of all objects in random order. For LTM trials, object presentation and test were separated by an unrelated memory task that lasted about 60 s. The task was designed to engage WM so that when the object colors were tested, they would have to be retrieved from LTM.

In Experiments 1 and 2, as depicted on the left panel of Fig. 1B, the distractor task consisted of a continuous orientation reproduction task. Participants worked through 10 trials. Each trial started with an 800 ms fixation cross, followed by four pointy black triangles shown for 400 ms on a white background, arranged in an imaginary cross centered on the screen. After a 1-s delay, one random triangle appeared in a random orientation. Participants used the mouse to adjust its orientation to the one they remembered seeing in that position and confirmed their response with a click. In Experiment 1, a blank screen followed for 2 s before the next trial. In Experiment 2 feedback was provided for 300 ms in the form of face icons: a green happy icon face appeared if the absolute error between the probe and the response was below 45°, a neutral yellow icon face if the error was between 46° and 60°, or a red sad icon face if the error exceeded 60°. This feedback was introduced with the aim of encouraging participant engagement and thereby enhancing the effectiveness of the distractor task in disrupting residual WM traces, as set size was reduced to three objects.

In Experiment 3, as depicted on the right panel of Fig. 1B, the distractor task consisted of the sequential presentation of four words^2^, each colored with one of the 360 hues from the color wheel described previously. Participants completed five trials. A trial started with an 800-ms fixation cross, followed by the words presented each for 1 s with an ISI of 500 ms. After all words were presented, one of two possible tests followed at random: a color-reproduction or a six-alternative forced-choice (6-AFC) test. In the color reproduction test, one randomly selected memory word appeared at the center of the screen in gray, surrounded by a gray wheel. Participants were asked to reproduce the color associated with the word as accurately as possible. By moving the mouse around the wheel, they could continuously adjust the color of the word, which was determined by the position of the mouse on the wheel. The response was locked with a mouse click. In the 6-AFC test, a colored square served as a cue, and participants were asked to click on the word associated with that color. They had to choose between six words presented in a random order in a row of boxes below the cue: the correct word, two trial lures (words presented in the trial but not associated with that color), and three new lures (two new adjectives and one color label not presented in that trial).

Finally, during the memory test in both tasks (Fig. 1C), all the objects were tested sequentially in random order. Each object appeared in gray at the center of the screen, surrounded by a gray wheel. Participants were instructed to reproduce the color of the object as accurately as possible by moving the mouse over the wheel. The color was determined by the current mouse position in the gray wheel and became instantly visible as the color of the object. By moving the mouse around the wheel, participants continuously adjusted the color of the object until they were satisfied and confirmed their selection with a mouse click.

### Data analysis

All analyses were implemented in R (R Core Team, 2023). For a first analysis, we submitted our data to Bayesian hierarchical linear regression models to predict the recall error—defined as the absolute distance in degrees (from 0° to 180°) between the response and the probed object’s color. The models were run with the *brms* package (Bürkner, 2017) and included three fixed-effect predictors: consolidation time, memory system (WM vs. LTM) and experiment, and their interactions. We fitted three separate models, one for each pair-wise comparison of two experiments. Each model included a random intercept, random slopes for consolidation time and memory system, and their interaction.

For model fitting, recall error was *z*-standardized. Consolidation time, originally measured in ms with six levels (300, 550, 1,050, 2,050, 5,050, and 8,050 ms), was transformed into equidistant values (−2.5, −1.5, −0.5, 0.5, 1.5, and 2.5, respectively) to facilitate model estimation. This ensured that the predictor was centered on zero while preserving its ordinal nature. Memory system (WM and LTM) and experiment were contrast-coded (−1, 1), allowing estimates to be centered on zero, too.

The fitting algorithm uses Markov chain Monte Carlo (MCMC) chains to estimate model parameters. We fitted each model with four chains, each consisting of 16,000 iterations after 1,000 warmup adaptation steps. For regression coefficients, we used Cauchy priors with a .5 scale. Chain convergence was assessed by visual inspection of the R-hat diagnostic, whose estimated value should not be larger than 1.05 to indicate that chains have converged (Vehtari et al., 2021).

We computed Bayes factors (BFs) with the *bayestestR* package (Makowski et al., 2019) that uses the Savage–Dickey Density Ratio (Wagenmakers et al., 2010). This ratio is calculated between the prior and the posterior distributions of a parameter of interest, evaluated at the parameter value of the null hypothesis (usually 0). Bayesian inference assesses the strength of evidence in favor or against a model, given the data (Kruschke & Liddell, 2018). For example, to gauge the evidence for a particular fixed effect, we compare a model including the effect of interest (Model 1) against a model excluding that effect (Model 0). The resulting BF is referred to as BF_10_. The BF should be interpreted continuously, with larger values indicating stronger evidence for Model 1 relative to Model 0. For example, a BF_10_ = 8 indicates that the data are eight times more likely on the assumption of Model 1 than on the assumption of Model 0.

Second, to more directly address the question whether the time-course of consolidation is the same or different between WM and LTM, we modelled and compared the parameters describing the consolidation curves in WM and LTM of each experiment by fitting a time-accuracy function described by the following formula:

$$RE= 90-\left(90-a\right)\bullet \left(1- {e}^{-r\bullet \left(t-{t}_{0}\right)}\right).$$

RE refers to the recall error, and *t* to the consolidation time. The recall error at *t* = 0 was set at 90°, as a recall error of this size reflects chance-level performance. Parameter *t*_*0*_ reflects the minimum amount of consolidation time needed to perform above chance. We consider this to reflect the *onset* time of consolidation. Parameter *r* represents the rate at which recall error decreases towards an asymptotic performance level. Finally, parameter *a* reflects the lower asymptote corresponding to the asymptotic level of recall error reached if unlimited consolidation time was provided. To ensure that the estimate of the onset time (*t*_*0*_) remained between 0 and the maximal consolidation time, we re-scaled the consolidation time to values between 0 and 1 by dividing all times by the maximum time (i.e., 8,050 ms). This enabled us to estimate the onset time on the logit scale as a normally distributed variable, which is transformed into *t*_*0*_ through the logistic function and ensured that *t*_*0*_ is constrained between 0 and 1.

To simultaneously estimate the consolidation-rate parameters for WM and LTM, we entered memory system (WM vs. LTM) as an indicator variable in the model. The indicator variable chooses one or the other set of model parameters for predicting the data, depending on the memory task. We fitted the model in a hierarchical Bayesian framework in *brms*, including random slopes and random intercepts as a function of participant for each model parameter.

We fitted the model with four chains, each one consisting of 20,000 iterations after 2,000 warmup adaptation steps, and thinning = 5. Normal prior distributions with means of 35 (*SD* = 10), 20 (*SD* = 7.5) and −2 (*SD* = 1.5) were used for the fixed effects on the *a*, *r* and logit (*t*_*0*_) parameters, respectively. Student’s* t* prior distributions with a scale of 3 were used for the random slopes of all the parameters. Chain convergence was assessed by visual inspection of the chains and by the R-hat diagnostic. Model parameters were evaluated by the mean and the 95% highest density interval (HDI) of their posterior. The HDI describes the interval in which the true value of the parameter lies with a posterior probability of .95.

## Results

**Fig. 2:**
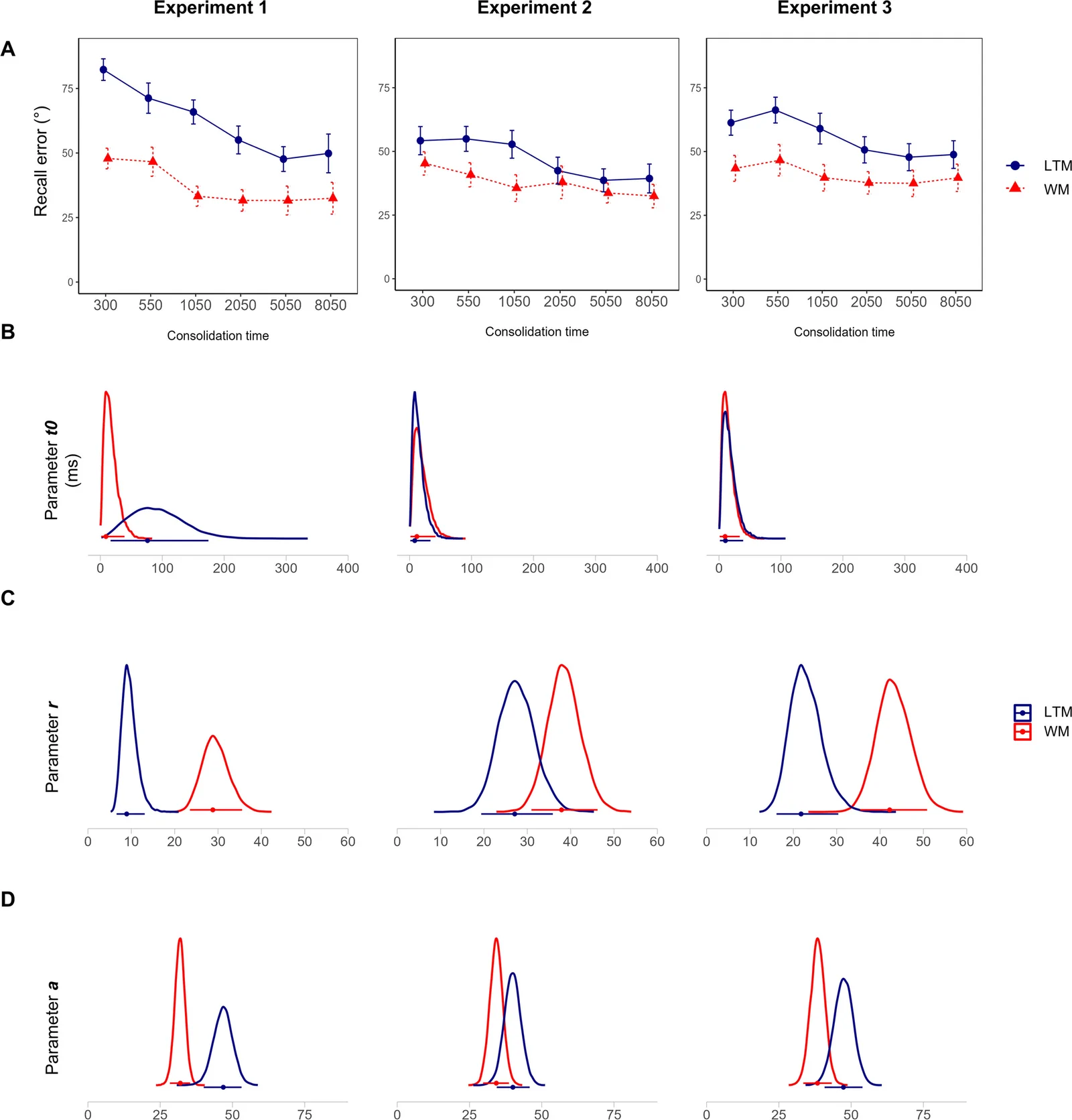
Results of Experiments 1, 2, and 3. **A** Recall error as a function of consolidation time and memory system. **B, C,** and **D** Posterior of the WM and LTM parameters for the onset time (*t*_*0*_), rate (*r*), and asymptote (*a*), respectively. (Color figure online)

Recall error as a function of consolidation time for WM and LTM across experiments is depicted in Fig. 2A. Across all experiments, the error decreased as more free time was available to consolidate the representations, following distinct trajectories for WM and LTM. As shown in Table 1, this is supported by strong evidence for main effects of consolidation time, memory system, and their interaction. These effects were consistent across the three experiments, as there was credible evidence *against* a three-way interaction between memory system, consolidation and experiment for all the models.

**Table 1:** Bayes factors of the main effects and interactions between experiments

	Experiment 1 vs 2		Experiment 1 vs 3		Experiment 2 vs 3	
Effect	BF_10_	BF_01_	BF_10_	BF_01_	BF_10_	BF_01_
Memory system	**6.76 × 10**^**10**^	—	**4.12 × 10**^**14**^	—	**2.23 × 10**^**8**^	--
Consolidation time	**5.79 × 10**^**5**^	—	**1.18 × 10**^**5**^	—	**1.67 × 10**^**3**^	--
Experiment	—	**19.23**	—	**20.40**	—	**17.85**
Memory system × Consolidation time	**82**	—	**828.51**	—	**397.68**	--
Memory system × Experiment	**2.31 × 10**^**3**^	—	**13.99**	—	—	**7.09**
Consolidation time × Experiment	—	**6.32**	—	1.08	—	**27.77**
Memory system × Consolidation time × Experiment	—	**12.82**	—	**40**	—	**58.82**

BFs > 3 that support or are against an effect are printed in **bold**

Our analysis revealed evidence *against* an interaction between consolidation time and experiment, showing that the effect of consolidation time replicates across the experiments. We obtained evidence that the effect of memory system differed between Experiment 1 and Experiments 2 and 3, but not between Experiments 2 and 3 (see Memory × Experiment interaction in Table 1). This reflects a set size effect in the LTM task: performance was credibly worse when participants had to consolidate and later retrieve 20 objects (Experiment 1) compared with 3 objects (Experiments 2 and 3).

The consolidation-time accuracy function provides a more precise description of the dynamics of consolidation. Figure 2B, C, and D show the posterior probability distributions of the model parameters describing the time-accuracy curves for WM and LTM: onset (*t0*), rate (*r*), and lower asymptote (*a*).

Regarding the onset of consolidation (Fig. 2B), the model estimated an earlier onset for WM compared to LTM in Experiment 1 (mean: WM = 17.3 ms, LTM = 91.9 ms) and Experiment 3 (mean: WM = 15.5 ms, LTM = 17.2 ms), but a slightly earlier onset for LTM in Experiment 2 (mean WM = 19 ms, LTM = 14.7 ms). However, in all three experiments, the 95% HDIs of the posterior difference distributions included zero (E1: 97.7% < 0 < 2.3% ; E2: 39.3%< 0 < 60.7%; and E3: 55.7% < 0 < 44.3%), indicating that the differences are not unambiguous, particularly in Experiments 2 and 3, where the onset estimates overlap substantially.^3^

For the rate, the results indicated that consolidation progresses at a faster rate in WM than in LTM in all three experiments. This difference was more strongly supported in Experiments 1 and 3, where the posterior distribution of the difference between the two rates lay more than 99% to one size of zero. The evidence was weaker in Experiment 2, where the posterior of the differences had more mass around zero (E2: 2.9% < 0 < 97.1%).

Lastly, consistent throughout the experiments, the asymptotic level of recall error was lower in WM than LTM. However, this difference was only unambiguous in Experiment 1, where the posterior difference was more than 99% on one side of zero. In Experiments 2 and 3 the posteriors of the difference were less conclusively away from zero (E2: 93.8% < 0 < 6.2%; E3: 98.3% < 0 < 1.7%). Descriptively, as observed in Fig. 2A, the time to reach asymptotic performance in WM across the three experiments aligns with previous estimates of the duration of short-term consolidation, reaching a plateau at around 1,000 ms (Nieuwenstein & Wyble, 2014; Ricker & Hardman, 2017). In contrast, LTM followed a slower trajectory. In Experiment 1, the error decreased gradually, reaching an asymptote at around 5,000 ms. In Experiments 2 and 3, LTM plateaued earlier—at around 2,000 ms.

**Fig. 3:**
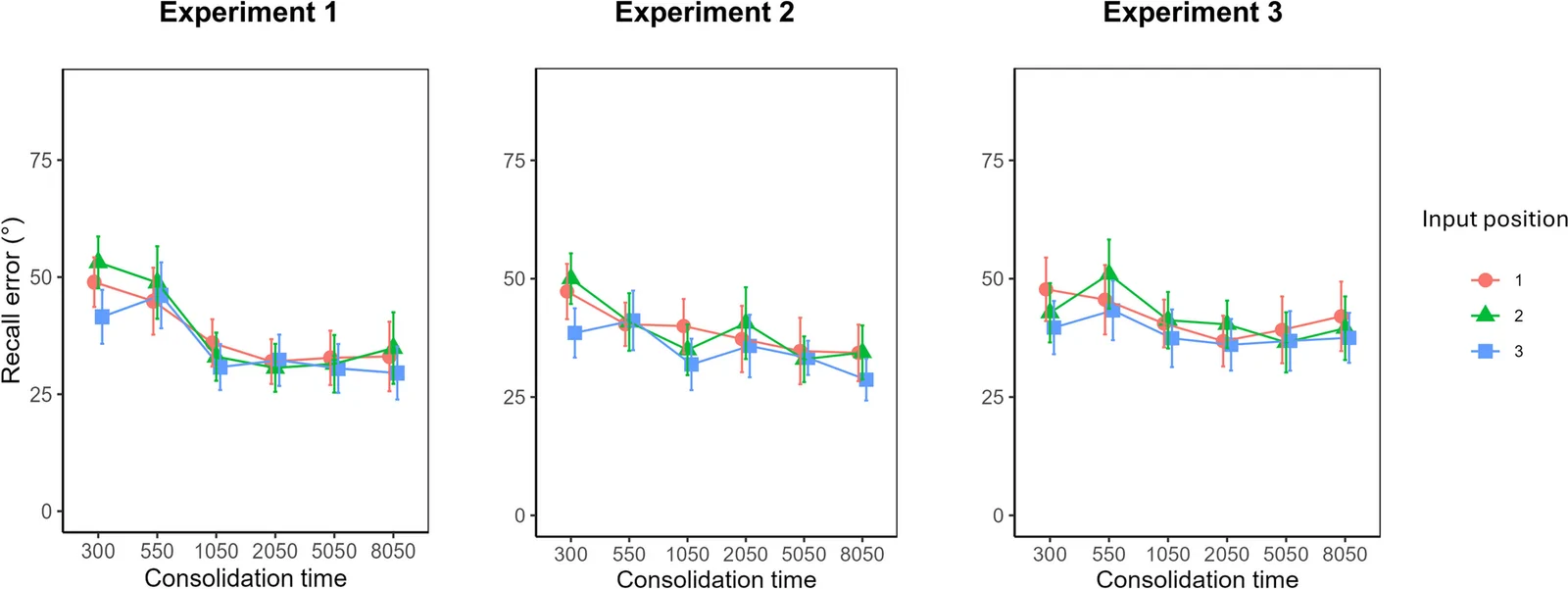
Recall error in the WM task for each serial input position as a function of consolidation time across experiments. Dots represent the sample mean and error bars show the 95% within-subjects confidence intervals. (Color figure online)

Previous research has suggested that short-term consolidation is a *ballistic* process, such that while one representation is being consolidated in WM, no other consolidation process can start (Ricker & Hardman, 2017). This predicts that the consolidation of a second target is impaired if it is presented shortly after the first target, because it appears in the time window when the first target is being consolidated (Lagroix et al., 2012). Because the first item is always fully consolidated, it should be remembered well irrespective of consolidation time. Contrary to this prediction, performance in our experiments improved for all items as a function of consolidation time, irrespective of their serial input (see Fig. 3), which is inconsistent with the conceptualization of consolidation as a ballistic process.

## General discussion

The present work examined the time course of establishing a consolidated representation in WM and in LTM by manipulating the free time following each memory object in a color-reproduction task. Across three experiments, we demonstrated that longer free time between items improved performance, but the consolidation time course differed between memory systems.

WM consolidation reached asymptote at around 1000 ms, in agreement with previous estimates of the duration of short-term consolidation. LTM consolidation progresses more slowly. This pattern was more robust in Experiments 1 and 3, where task differences allowed for a clearer dissociation between WM and LTM.

In Experiment 1, LTM trials required participants to encode and retrieve 20 items—unambiguously exceeding WM capacity–—resulting in a slower and prolonged consolidation trajectory. This yielded clear differences between LTM and WM in both consolidation rate and asymptotic performance. In contrast, Experiments 2 and 3 included only three items in both memory tasks, which in turn led to faster consolidation in LTM compared with Experiment 1.

In Experiment 3, but not in Experiment 2, there was a credible difference in the rate parameter between memory systems. In Experiment 2, there is the possibility that the distractor task did not fully disrupt WM maintenance of the colored objects during the delay, because the distractor task involved orientations, which cause little if any interference with object-color conjunctions (Lin & Oberauer, 2022; Oberauer et al., 2012). This could have allowed participants to rely on a mixture of WM and LTM traces at retrieval, which could explain why performance in the LTM test was barely worse than in WM, and why there were only small, statistically ambiguous differences in the rate and asymptotic parameters. Support for this possibility comes from a version of Experiment 2 that used unique objects in each trial (Experiment S1 in the Online Supplementary Materials), in which we found no evidence for an interaction between memory system and consolidation time and no conclusive evidence for differences in any parameter of the time-accuracy model. This was likely the case because the unique representations, which minimized proactive interference, allowed the mixture of WM and LTM traces to be useful in both the immediate and delayed tests.

In Experiment 3, we therefore modified the distractor task to include visual and verbal color information, so that it interferes with both visual representations and with possible verbal representations of the colored objects. In this way, we could ensure that the LTM test in Experiment 3 was not contaminated by contributions from WM. Consequently, compared to Experiment 2, Experiments 1 and 3 more clearly dissociated the creation of WM and LTM representations. Both experiments show unambiguously that LTM consolidation progresses at a lower rate than WM. In other words, LTM traces take longer to be transferred to a robust format, and therefore, there are different processes contributing to the creation of short and long-term representations.

As the onset of WM and LTM consolidation differ only very little in time, the two processes could run independently in parallel. Alternatively, they could operate simultaneously in a cascade fashion (McClelland, 1979), where information, once consolidated in WM, is transferred to LTM. Hence, LTM consolidation starts as soon as some information becomes available from WM consolidation. Another possibility is that short-term consolidation initially establishes both WM and LTM alike, but for LTM, additional slower processes further contribute to the formation of stable memory traces, particularly when the amount of information to maintain increases. One such mechanism could be elaboration, a process where information is more deeply encoded by activating semantic properties and creating meaningful associations (Craik & Tulving, 1975). Elaborative strategies, such as mental imagery or sentence generation, are highly effective for episodic LTM but not for WM (Bartsch et al., 2018; Bartsch & Oberauer, 2021; Thalmann et al., 2019). Although we have no direct evidence that people were engaging in such strategies, we have no evidence against that either. Future studies may need to tease apart these possibilities.

The slower consolidation time-course for episodic LTM provides evidence that contributions from newly formed episodic LTM traces to WM performance are more plausible if more time to process the information is provided. However, in typical WM tasks, the amount of information that people can remember is severely limited (Cowan, 2001). Therefore, the contribution of episodic LTM cannot be unrestricted. One explanation for a limited contribution of episodic LTM to WM is that the former is susceptible to proactive interference, whereas WM is not (Cowan et al., 2005; Oberauer, 2009; Oberauer et al., 2017; Wickens et al., 1963). Accordingly, when WM representations are sufficient to solve the tasks, people tend to rely exclusively on them (Bartsch & Shepherdson, 2022).

Furthermore, our data across all three experiments is inconsistent with the claim that consolidation is a ballistic process. When not enough time was provided to create a stable WM trace, consolidation was disrupted by presentation of the subsequent object. This has been referred to as *“retroactive interference”* with a consolidation process that has already been initiated (Nieuwenstein & Wyble, 2014). Our results could also be interpreted with a single resource consolidation account (Navon & Miller, 2002), in which allocation of a resource can be shared concurrently among items. With little time to fully consolidate each item, consolidation of multiple items occurs in parallel, which increases the competition for the resource and impairs the creation of stable memory representations. Longer ISIs increase the likelihood of fully consolidating each item by reducing the temporal overlap between consolidation processes and the competition for the resource.

For a similar reason, our findings are also difficult to reconcile with the assumption that encoding into memory depletes an encoding resource that takes time to replenish (Mızrak & Oberauer, 2021; Popov & Reder, 2020). On that hypothesis, longer ISI allows the encoding resource to be replenished, thereby enabling stronger encoding of subsequent items. This would lead to a benefit of longer time that is nonexistent for the first item and increases with every additional item presented. Our observation that the effect of time did not interact with serial position contradicts that prediction.

An alternative explanation of our findings could be that increasing the ISI entails that the memoranda were more temporally spaced, which in turn may reduce their confusability. This is what is predicted by a temporal distinctiveness theory of memory (Brown et al., 2007; Brown & Lewandowsky, 2010; Ecker et al., 2015). According to this theory, memories are organized, among other things, along a psychological time dimension. Assuming that time acts as a retrieval cue, items that occupy nearby locations on the time dimension will tend to be confused with one another. As time passes, psychological time is logarithmically compressed. Hence, the temporal distinctiveness of an item is the ratio between the elapsed time since the presentation of to-be-remembered item to the moment of test (i.e., the retention interval) and the temporal distance to other potentially interfering items (determined by the ISI). The lower the ratio, the higher the distinctiveness, and hence, the probability that an item will be recalled (Brown et al., 2009). As we varied the consolidation time through the ISI, the conditions with longer consolidation time had higher temporal distinctiveness. We addressed this confound in Experiment S2 (see the Online Supplementary Materials). In brief, our results provided no evidence for a contribution of temporal distinctiveness, and strong evidence in favor of the consolidation hypothesis: Perceptual traces are transformed gradually into durable representations as a function of time provided after each object.

Taken together, our findings demonstrate that the time-course to create stable WM and LTM representations differs, likely reflecting two different consolidation processes. Time-accuracy functions revealed that these processes can run partially in parallel, with WM proceeding at a faster rate and asymptoting at a lower average error. Consequently, our results suggest that LTM traces may contribute to WM when sufficient time is given for the consolidation of episodic LTM representations.

## Data Availability

The data can be accessed on the Open Science Framework: https://osf.io/t8e3p/?view_only=1064bf607a724f47b77aeacb2d158fb3.

## Footnotes

1 In principle, one could entertain the possibility of a faster rate of consolidation in LTM than WM, but this would not be theoretically reasonable because WM is a system that serves more immediate demands, which requires a quick turnover of representations to guide our thoughts and actions.

2 The words were adjectives selected from an English list of traits (ELot; Britz et al., 2022) with an average frequency of 3.63, and 14 color labels (e.g., “blue”). A trial list included 2 adjectives and 2 color labels. All the words were translated to German.

3 Posterior difference distributions are posterior probability distributions of the difference between two parameters (e.g., the onset of consolidation in WM and in LTM tests); they are obtained by taking the difference between the two parameters in every MCMC chain.

## Code availability

The scripts for the analysis and experiments can be accessed on the Open Science Framework: https://osf.io/t8e3p/?view_only=1064bf607a724f47b77aeacb2d158fb3.

## References

[CR1] Atkinson, R. C., & Shiffrin, R. M. (1968). Human memory: A proposed system and its control processes. *Psychology of Learning and Motivation,**2*, 89–195. 10.1016/S0079-7421(08)60422-3

[CR2] Bartsch, L. M., & Oberauer, K. (2021). The effects of elaboration on working memory and long-term memory across age. *Journal of Memory and Language,**118*, Article 104215. 10.1016/j.jml.2020.104215

[CR3] Bartsch, L. M., & Oberauer, K. (2023). The contribution of episodic long-term memory to working memory for bindings. *Cognition,**231*, Article 105330. 10.1016/j.cognition.2022.105330 36436446

[CR4] Bartsch, L. M., & Shepherdson, P. (2022). Freeing capacity in working memory (WM) through the use of long-term memory (LTM) representations. *Journal of Experimental Psychology: Learning, Memory, and Cognition,**48*(4), Article 465. 10.1037/xlm0001024 34292052

[CR5] Bartsch, L. M., Singmann, H., & Oberauer, K. (2018). The effects of refreshing and elaboration on working memory performance, and their contributions to long-term memory formation. *Memory & Cognition,**46*(5), 796–808. 10.3758/s13421-018-0805-9 29557069

[CR6] Britz, S., Rader, L., Gauggel, S., & Mainz, V. (2022). An English list of trait words including valence, social desirability, and observability ratings. *Behavior Research Methods,**55*(5), 2669–2686. 10.3758/s13428-022-01921-5 PMC10439032 35962307

[CR7] Brown, G. D. A., Neath, I., & Chater, N. (2007). A temporal ratio model of memory. *Psychological Review,**114*(3), 539–576. 10.1037/0033-295X.114.3.539 17638496

[CR8] Brown, G. D. A., & Lewandowsky, S. (2010). Forgetting in memory models: Arguments against trace decay and consolidation failure. In S. Della Sala (Ed.), *Forgetting* (pp. 49–75). Psychology Press.

[CR9] Brown, G. D. A., Vousden, J. I., & McCormack, T. (2009). Memory retrieval as temporal discrimination. *Journal of Memory and Language,**60*(1), 194–208. 10.1016/j.jml.2008.09.003

[CR10] Bürkner, P.-C. (2017). brms: An R package for Bayesian multilevel models using Stan. *Journal of Statistical Software,**80*, 1–28.

[CR11] Cotton, K., & Ricker, T. J. (2021). Working memory consolidation improves long-term memory recognition. *Journal of Experimental Psychology: Learning, Memory, and Cognition,**47*(2), 208–219. 10.1037/xlm0000954 32833468

[CR12] Cotton, K., & Ricker, T. J. (2022). Examining the relationship between working memory consolidation and long-term consolidation. *Psychonomic Bulletin & Review*. 10.3758/s13423-022-02084-2 35357669

[CR13] Cowan, N. (2001). The magical number 4 in short-term memory: A reconsideration of mental storage capacity. *Behavioral and Brain Sciences,**24*(1), 87–114. 10.1017/S0140525X01003922 11515286

[CR14] Cowan, N. (2008). What are the differences between long-term, short-term, and working memory? In *Progress in brain research* (Vol. 169, pp. 323–338). Elsevier. 10.1016/S0079-6123(07)00020-9 PMC2657600 18394484

[CR15] Cowan, N., Johnson, T. D., & Scott Saults, J. (2005). Capacity limits in list item recognition: Evidence from proactive interference. *Memory,**13*(3/4), 3–4. 10.1080/09658210344000206 15952261

[CR16] Craik, F. I. M., & Tulving, E. (1975). Depth of processing and the retention of words in episodic memory. *Journal of Experimental Psychology: General,**104*(3), 268–294. 10.1037/0096-3445.104.3.268

[CR17] Ecker, U. K. H., Brown, G. D. A., & Lewandowsky, S. (2015). Memory without consolidation: Temporal distinctiveness explains retroactive interference. *Cognitive Science,**39*(7), 1570–1593. 10.1111/cogs.12214 25556982

[CR18] Hartshorne, J. K., & Makovski, T. (2019). The effect of working memory maintenance on long-term memory. *Memory & Cognition,**47*(4), 749–763. 10.3758/s13421-019-00908-6 PMC6663308 31073790

[CR19] Henninger, F., Shevchenko, Y., Mertens, U. K., Kieslich, P. J., & Hilbig, B. E. (2021). Lab. Js: A free, open, online study builder. *Behavior Research Methods*. 10.3758/s13428-019-01283-5 PMC9046347 34322854

[CR20] Jolicœur, P., & Dell’Acqua, R. (1998). The demonstration of short-term consolidation. *Cognitive Psychology,**36*(2), 138–202. 10.1006/cogp.1998.0684 9721199

[CR21] Kruschke, J. K., & Liddell, T. M. (2018). The Bayesian New Statistics: Hypothesis testing, estimation, meta-analysis, and power analysis from a Bayesian perspective. *Psychonomic Bulletin & Review,**25*(1), 178–206. 10.3758/s13423-016-1221-4 28176294

[CR22] Lagroix, H. E. P., Spalek, T. M., Wyble, B., Jannati, A., & Di Lollo, V. (2012). The root cause of the attentional blink: First-target processing or disruption of input control? *Attention, Perception, & Psychophysics,**74*(8), 1606–1622. 10.3758/s13414-012-0361-5 22893006

[CR23] Lin, H.-Y., & Oberauer, K. (2022). An interference model for visual working memory: Applications to the change detection task. *Cognitive Psychology,**133*, Article 101463. 10.1016/j.cogpsych.2022.101463 35151184

[CR24] Makowski, D., Ben-Shachar, M. S., & Lüdecke, D. (2019). bayestestR: Describing effects and their uncertainty, existence and significance within the Bayesian framework. *Journal of Open Source Software,**4*(40), Article 1541.

[CR25] McCabe, D. P. (2008). The role of covert retrieval in working memory span tasks: Evidence from delayed recall tests. *Journal of Memory and Language,**58*(2), 480–494. 10.1016/j.jml.2007.04.004 PMC2715014 19633737

[CR26] McClelland, J. L. (1979). On the time relations of mental processes: An examination of systems of processes in cascade. *Psychological Review,**86*(4), 287–330.

[CR27] Mızrak, E., & Oberauer, K. (2021). What is time good for in working memory? *Psychological Science,**32*(8), 1325–1337. 10.1177/0956797621996659 PMC13038132 34309420

[CR28] Navon, D., & Miller, J. (2002). Queuing or sharing? A critical evaluation of the single-bottleneck notion. *Cognitive Psychology,**44*(3), 193–251. 10.1006/cogp.2001.0767 11971632

[CR29] Nieuwenstein, M., & Wyble, B. (2014). Beyond a mask and against the bottleneck: Retroactive dual-task interference during working memory consolidation of a masked visual target. *Journal of Experimental Psychology: General,**143*(3), 1409–1427. 10.1037/a0035257 24364683

[CR30] Oberauer, K. (2009). Design for a working memory. In *Psychology of Learning and Motivation* (Vol. 51, pp. 45–100). Elsevier. 10.1016/S0079-7421(09)51002-X

[CR31] Oberauer, K., & Awh, E. (2022). Is there an activity-silent working memory? *Journal of Cognitive Neuroscience,**34*(12), 2360–2374. 10.1162/jocn_a_01917 PMC10158524 36122353

[CR32] Oberauer, K., Awh, E., & Sutterer, D. W. (2017). The role of long-term memory in a test of visual working memory: Proactive facilitation but no proactive interference. *Journal of Experimental Psychology: Learning, Memory, and Cognition,**43*(1), 1–22. 10.1037/xlm0000302 PMC5209290 27685018

[CR33] Oberauer, K., Lewandowsky, S., Farrell, S., Jarrold, C., & Greaves, M. (2012). Modeling working memory: An interference model of complex span. *Psychonomic Bulletin & Review,**19*(5), 779–819. 10.3758/s13423-012-0272-4 22715024

[CR34] Popov, V., & Reder, L. M. (2020). Frequency effects on memory: A resource-limited theory. *Psychological Review,**127*(1), 1–46. 10.1037/rev0000161 31524424

[CR35] R Core Team. (2023). *R: A language and environment for statistical computing.* R Foundation for Statistical Computing.

[CR36] Ricker, T. J., & Cowan, N. (2014). Differences between presentation methods in working memory procedures: A matter of working memory consolidation. *Journal of Experimental Psychology: Learning, Memory, and Cognition,**40*(2), 417–428. 10.1037/a0034301 PMC4056671 24059859

[CR37] Ricker, T. J., & Hardman, K. O. (2017). The nature of short-term consolidation in visual working memory. *Journal of Experimental Psychology: General,**146*(11), 1551. 10.1037/xge0000346 28703619

[CR38] Ricker, T. J., Nieuwenstein, M. R., Bayliss, D. M., & Barrouillet, P. (2018). Working memory consolidation: Insights from studies on attention and working memory: An overview of working memory consolidation. *Annals of the New York Academy of Sciences,**1424*(1), 8–18. 10.1111/nyas.13633 29635694

[CR39] Sobrinho, N. D., & Souza, A. S. (2023). The interplay of long-term memory and working memory: When does object-color prior knowledge affect color visual working memory? *Journal of Experimental Psychology: Human Perception and Performance,**49*, 236–262. 10.1037/xhp0001071 36480376

[CR40] Souza, A. S., & Oberauer, K. (2017). Time to process information in working memory improves episodic memory. *Journal of Memory and Language,**96*, 155–167.

[CR41] Stevanovski, B., & Jolicœur, P. (2007). Visual short-term memory: Central capacity limitations in short-term consolidation. *Visual Cognition,**15*(5), 532–563. 10.1080/13506280600871917

[CR42] Sutterer, D. W., & Awh, E. (2016). Retrieval practice enhances the accessibility but not the quality of memory. *Psychonomic Bulletin & Review,**23*(3), 831–841. 10.3758/s13423-015-0937-x PMC4808484 26404635

[CR43] Thalmann, M., Souza, A. S., & Oberauer, K. (2019). Revisiting the attentional demands of rehearsal in working-memory tasks. *Journal of Memory and Language,**105*, 1–18.

[CR44] Unsworth, N., & Engle, R. (2006). A temporal–contextual retrieval account of complex span: An analysis of errors☆. *Journal of Memory and Language,**54*(3), 346–362. 10.1016/j.jml.2005.11.004

[CR45] Unsworth, N., & Engle, R. W. (2006). Simple and complex memory spans and their relation to fluid abilities: Evidence from list-length effects. *Journal of Memory and Language,**54*(1), 68–80. 10.1016/j.jml.2005.06.003

[CR46] Unsworth, N., & Engle, R. W. (2007). The nature of individual differences in working memory capacity: Active maintenance in primary memory and controlled search from secondary memory. *Psychological Review,**114*(1), 104–132. 10.1037/0033-295X.114.1.104 17227183

[CR47] Vehtari, A., Gelman, A., Simpson, D., Carpenter, B., & Bürkner, P.-C. (2021). Rank-normalization, folding, and localization: An improved R for assessing convergence of MCMC (with discussion). *Bayesian Analysis,**16*(2), 667–718.

[CR48] Wagenmakers, E.-J., Lodewyckx, T., Kuriyal, H., & Grasman, R. (2010). Bayesian hypothesis testing for psychologists: A tutorial on the Savage–Dickey method. *Cognitive Psychology,**60*(3), Article 3. 10.1016/j.cogpsych.2009.12.001 20064637

[CR49] Wickens, D. D., Born, D. G., & Allen, C. K. (1963). Proactive inhibition and item similarity in short-term memory. *Journal of Verbal Learning and Verbal Behavior,**2*(5–6), Article 5–6. 10.1016/S0022-5371(63)80045-6

